# ASSOCIATION BETWEEN INCREASED CAREGIVER BURDEN AND MENTAL HEALTH DURING THE COVID-19 PANDEMIC IN JAPAN

**DOI:** 10.1093/geroni/igac059.2056

**Published:** 2022-12-20

**Authors:** Isuzu Nakamoto, Hiroshi Murayama, Mai Takase, Yoko Muto, Tami Saito, Takahiro Tabuchi

**Affiliations:** Tohoku University, Tokyo, Tokyo, Japan; Tokyo Metropolitan Institute of Gerontology, Tokyo, Tokyo, Japan; The University of Tokyo, Tokyo, Tokyo, Japan; Tokyo Metropolitan Institute of Gerontology, Tokyo, Tokyo, Japan; National Center for Geriatrics and Gerontology, Obu, Aichi, Japan; Osaka International Cancer Institute, Osaka, Osaka, Japan

## Abstract

During the COVID-19 pandemic, informal caregivers’ mental health was more deteriorated than non-caregivers. The objective of this study was to examine the association between increased caregiver burden and severe psychological distress (SPD) during the pandemic. We used cross-sectional data from a nationwide internet survey conducted between August and September 2020 in Japan. Of 25,482 participants aged 15–79 years, 1,920 informal caregivers were analyzed (mean age, 52.3 ± 15.9 years; men, 48.8%). SPD was defined as the Kessler 6 Scale (K6) ≥ 13. The self-rated change in caregiver burden was measured with the single question item. A binary logistic regression analysis was used to examine the association between SPD and increased caregiver burden, adjusted for demographic, socioeconomic, health, and caregiving variables. To examine the differential association between increased caregiver burden and SPD, interaction terms were added and binary logistic regression was separately conducted in all variables. Of total caregivers, 56.7% reported the increased caregiver burden and 19.3% were SPD. Increased caregiver burden was significantly associated with SPD, with an adjusted odds ratio 1.90 (95% confidence interval 1.37–2.66). The association between increased caregiver burden and SPD was stronger among married caregivers, with disease treatment, and with their care-receiver’s care need level 1–2 (Japanese long term care service levels). This study demonstrated that increased caregiver burden was associated with SPD during the pandemic. Therefore, measures against mental health for the caregivers with increased caregiver burden need to be implemented immediately.

